# Case Report: Panic attack induced by albendazole in a post-PCI patient

**DOI:** 10.3389/fpsyt.2026.1915974

**Published:** 2026-08-28

**Authors:** Rijian Wang, Qiuyan Huang, Li Zhang

**Affiliations:** 1 Department of Clinical Pharmacy, People's Hospital of Guangxi Zhuang Autonomous Region (Guangxi Academy of Medical Sciences), Nanning, Guangxi, China; 2 School of Pharmacy, Guangxi University of Chinese Medicine, Nanning, Guangxi, China; 3 Department of Pharmacy, Ping Guo People’s Hospital, Baise, Guangxi, China

**Keywords:** adverse drug reaction, albendazole, case report, clonorchiasis, drug-induced panic attack, panic disorder, psychopharmacology

## Abstract

**Background:**

Albendazole is a widely used broad-spectrum anthelmintic whose recognized neuropsychiatric adverse effects are largely limited to encephalitis and demyelinating encephalopathy. Panic attack has not been previously reported as an adverse reaction to albendazole.

**Case presentation:**

A 59-year-old Chinese man with recent percutaneous coronary intervention for coronary artery disease was prescribed albendazole (600 mg/day for 7 days) for an incidentally detected *Clonorchis sinensis* infection. Within hours of his first dose, he developed recurrent, unpredictable panic attacks characterized by palpitations, chest pain, dyspnea, derealization, fear of dying, and a sense of losing control. These episodes met DSM-5 diagnostic criteria for panic attack (11 of 13 core symptoms) and were graded as Grade 2 according to the Common Terminology Criteria for Adverse Events version 5.0. Extensive cardiac, pulmonary, and psychiatric workup excluded alternative causes. The panic attacks resolved following albendazole discontinuation and combination anxiolytic–antidepressant therapy. The Naranjo adverse drug reaction probability score was 8, indicating a probable causal relationship.

**Conclusions:**

This case suggests that albendazole can probably induce acute panic attacks, expanding its neuropsychiatric adverse effect spectrum. In patients with heightened central nervous system vulnerability—such as those recovering from major cardiovascular procedures—the risk may be increased. For non-urgent anthelmintic indications in such patients, deferring treatment until clinical stabilization may be prudent.

## 1 Introduction

Albendazole, a benzimidazole-class broad-spectrum anthelmintic, is widely used for the treatment of intestinal helminthiasis including ascariasis, enterobiasis, and clonorchiasis (1). While generally well tolerated, its adverse drug reactions (ADRs) may involve the digestive, hematologic, and central nervous systems. Notably, neurotoxicity is well documented: serious neurological ADRs include encephalitis and demyelinating encephalopathy, which typically present with headache, dizziness, lethargy, and in severe cases, altered consciousness and quadriplegia (2, 3). Regulatory agencies have issued warnings regarding the risk of encephalitis associated with albendazole and other imidazole-class anthelmintics (3, 4), underscoring the clinical importance of recognizing its neurological ADR spectrum.

Panic attack, characterized by abrupt intense fear or discomfort with autonomic arousal, is predominantly a primary psychiatric disorder. Drug-induced panic attacks are relatively rare: a 30-year analysis of the French pharmacovigilance database identified antidepressants, mefloquine, isotretinoin, and corticosteroids as the main offending agents, yet antiparasitic drugs were conspicuously absent (5). Importantly, iatrogenic panic attacks mostly occur in patients without antecedent psychiatric history, are frequently underrecognized, and are rarely labeled in the prescribing information of suspected drugs (5). Although rare cases of albendazole-induced chronic anxiety and acute psychosis have been reported, no previous case of albendazole-induced acute panic attack has been described. To our knowledge, this is the first report of a DSM-5-defined acute panic attack induced by albendazole, highlighting a novel and potentially underrecognized adverse drug reaction.

## 2 Case presentation

### 2.1 Patient history and initial presentation

A 59-year-old Chinese man presented to our emergency department on November 10, 2025, with a 10-day history of recurrent, unpredictable panic-like episodes. He had undergone percutaneous coronary intervention (PCI) with a drug-eluting stent in the proximal left anterior descending artery on October 27, 2025, following an acute coronary syndrome. Routine stool examination during that admission incidentally detected *Clonorchis sinensis* ova. He was discharged on October 29 on dual antiplatelet therapy (aspirin 100 mg/day, clopidogrel 75 mg/day), atorvastatin 20 mg/day, sacubitril/valsartan, metoprolol succinate, and amlodipine. During the first 48 hours post-discharge (October 29–30), he remained asymptomatic on these medications alone.

On October 31, he was prescribed oral albendazole 600 mg/day (10 mg/kg) for clonorchiasis. Within hours of the first dose, he experienced a sudden onset of palpitations, chest tightness, dyspnea, derealization, an overwhelming fear of dying, and a sense of losing control. The attacks lasted several minutes, resolved spontaneously, and recurred unpredictably throughout the 7-day albendazole course. Anticipatory anxiety, agoraphobic avoidance, poor appetite, and insomnia developed progressively. A detailed clinical timeline is provided in **Table 1**.

**Table 1:** Clinical timeline of key events in the case.

Date (2025)	Event & key clinical findings
October 22	Presented to the emergency department with dizziness, headache, chest tightness, and chest pain of 3 days’ duration. Blood pressure 193/122 mm Hg. Initial assessment: acute coronary syndrome suspected;Managed with intravenous nitroglycerin and antihypertensives.
October 24	Admitted to the Department of cardiology; Coronary angiography confirmed significant stenotic lesion in the proximal left anterior descending artery
October 27	Percutaneous coronary intervention (PCI) performed; a 3.5 × 15 mm drug-eluting stent was deployed in the proximal left anterior descending artery. Dual antiplatelet therapy (aspirin 100 mg/day, clopidogrel 75 mg/day), atorvastatin 20 mg/day, and antihypertensive regimen (sacubitril/valsartan, metoprolol succinate, amlodipine) were initiated. mm
October 28	– Routine stool examination detected Clonorchis sinensis ova (0–1 per low-power field); clonorchiasis diagnosed incidentally.
October 29	– Discharged in stable cardiovascular condition. October 29–30: asymptomatic period (negative control) — no anxiety or panic symptoms reported while taking only cardiovascular medications.
October 31 (morning)	Initiated oral albendazole 600 mg/day (10 mg/kg) for *clonorchiasis* treatment.
October 31 (hours later)	First acute panic attack occurred, presenting with sudden palpitations, chest pain, dyspnea, derealization, fear of dying, and sense of losing control.
October 31 – November 6	Recurrent, unpredictable panic attacks persisted throughout the 7-day albendazole treatment course. Progressive development of anticipatory anxiety, agoraphobic avoidance, and insomnia.
November 6	Completed 7-day albendazole course; drug was discontinued. Panic attacks and anxiety symptoms continued to occur.
November 10	labor Re-presented to the emergency department with persistent anxiety and panic symptoms. Comprehensive workup (serial ECG, hs-cTnI, CK-MB, NT-proBNP, echocardiography, chest CT, arterial blood gas) excluded acute coronary syndrome, pulmonary embolism, and other organic causes. Panic attack confirmed, meeting 11 of 13 DSM-5 diagnostic criteria.
November 11	Admitted to the psychiatric ward. Alprazolam 0.8 mg twice daily was initiated for acute anxiolysis. Three additional panic attacks occurred during the first 3 days of hospitalization.
November 11–14	Pharmacotherapy was gradually intensified: duloxetine, buspirone, and eszopiclone were added and titrated. Panic attacks completely ceased by hospital day 3.
November 19	Discharged on a stable oral psychotropic regimen, with scheduled outpatient psychiatric follow-up for consolidation therapy.
Overall assessment	Naranjo ADR probability score = 8 (probable causal relationship). ADR graded as CTCAE v5.0 Grade 2 (moderate).

PCI, percutaneous coronary intervention; ECG, electrocardiogram; hs-cTnI, high-sensitivity cardiac troponin I; CK-MB, creatine kinase-MB; NT-proBNP, N-terminal pro-B-type natriuretic peptide; CT, computed tomography; ABG, arterial blood gas; CTCAE, Common Terminology Criteria for Adverse Events.

On re-presentation (November 10), the patient appeared anxious but was fully oriented to time, place, and person. Vital signs on arrival were as follows: heart rate 93 bpm, blood pressure 140/86 mm Hg, respiratory rate 18/min, and body temperature 36.5 °C. Cardiopulmonary examination revealed clear breath sounds bilaterally without rales or wheezes, regular heart rhythm with no murmurs, gallops, or pericardial friction rub, and no signs of congestive heart failure such as peripheral edema or jugular venous distension. Neurological examination was unremarkable: cranial nerves were intact, motor strength and tone were symmetric in all four limbs, deep tendon reflexes were normal, and no focal deficits or pathological reflexes were detected. Mental status examination demonstrated anxious affect, slightly accelerated speech rate, intact attention and memory, no hallucinations or delusions, and preserved insight into his symptoms.

### 2.2 Diagnostic assessment

Extensive workup was performed to exclude organic causes. Serial electrocardiograms, high-sensitivity cardiac troponin, creatine kinase-MB, N-terminal pro-B-type natriuretic peptide, and transthoracic echocardiography were all within normal limits, ruling out acute coronary syndrome, stent thrombosis, and structural heart disease. Chest CT showed only mild pneumonitis and focal fibrosis; pulmonary embolism was excluded. Arterial blood gas revealed a mild respiratory alkalosis (pH 7.45, PCO_2_ 31 mm Hg), consistent with hyperventilation during panic. Liver enzymes were normal, and blood glucose was 9.00 mmol/L, consistent with previously untreated hyperglycemia.

The primary diagnostic challenge in this case was distinguishing drug-induced panic attacks from acute cardiovascular events, given the patient’s recent PCI and presenting symptoms of chest pain and palpitations. Stepwise differential diagnosis was performed as follows:

1. Acute coronary syndrome/stent thrombosis: Ruled out by serial normal electrocardiograms, unchanged high-sensitivity cardiac troponin levels, and normal left ventricular function on echocardiography.
2. Pulmonary embolism: Excluded by the absence of typical risk factors and unremarkable chest CT findings.
3. Endocrine causes (e.g., hyperthyroidism, pheochromocytoma): Not supported by clinical presentation; further workup was not indicated as symptoms were time-locked to albendazole initiation.
4. Primary panic disorder: Less likely, as the patient had no prior psychiatric history, no family history of anxiety disorders, and a clear temporal relationship between symptom onset and drug exposure.
5. Acute stress reaction post-PCI: Although recent PCI represented a significant stressor, the patient remained asymptomatic for 48 hours after discharge on identical cardiovascular medications, making isolated post-procedural stress an unlikely sole cause.

Prognostically, drug-induced panic attacks generally have a favorable outcome when the offending agent is promptly discontinued and appropriate symptomatic treatment is initiated. However, delayed recognition may lead to progression to persistent panic disorder with agoraphobia, as observed in this patient.

The panic episodes met DSM-5 criteria for a panic attack (6), with 11 of the 13 core symptoms present (nausea/abdominal distress and chills/hot flushes were absent). According to the Common Terminology Criteria for Adverse Events version 5.0 (7), the adverse drug reaction was graded as Grade 2 (moderate), as it was recurrent, impaired daily functioning, and required pharmacologic intervention. The patient had no prior psychiatric history or family history of mental illness, and other concomitant cardiovascular drugs have no known association with panic attacks. A diagnosis of substance-induced panic attack (suspected agent: albendazole) with secondary insomnia was made. The Naranjo adverse drug reaction probability scale (8) yielded a score of 8, indicating a “probable” causal relationship (**Table 2**).

**Table 2:** Naranjo adverse drug reaction probability scale (albendazole-associated panic attack).

Assessment item	Score	Patient’s response	Points awarded
1. Are there previous conclusive reports on this reaction?	0/+1/+2	No rare reports of albendazole-induced panic attack	0
2. Did the adverse event appear after the suspected drug was administered?	0/+1/+2	Onset within 1 day of albendazole initiation	+2
3. Did the adverse reaction improve when the drug was discontinued?	0/+1/+2	Remitted after drug cessation + anti-anxiety treatment	+1
4. Did the adverse event reappear when the drug was readministered?	0/-1/+3	Not rechallenged	0
5. Are there alternative causes that could have caused the reaction?	0/-2/+2	All organic/psychiatric causes excluded	+2
6. Did the reaction reappear when a placebo was given?	0/-1/+1	No placebo challenge	0
7. Was the drug detected in toxic concentrations?	0/+1/+2	No toxic accumulation (normal lab tests)	0
8. Is the reaction more severe with increased dose?	0/+1	Standard dose (10 mg/kg/d)	0
9. Did the patient have a similar reaction previously?	0/+1	No prior panic attack history	+1
10. Was the adverse event confirmed by objective evidence?	0/+1/+2	Confirmed by DSM-5 + clinical observation	+2
Total Score			8
Causality Category			Probable

### 2.3 Therapeutic intervention and outcomes

Albendazole was discontinued upon completion of the 7-day course (November 6). Due to persistent panic attacks, the patient was referred to psychiatry and admitted on November 11.

Pharmacotherapy was initiated with alprazolam 0.8 mg twice daily, which provides rapid anxiolytic and sedative effects to control acute panic episodes and insomnia. Over the first three days of hospitalization, three further discrete panic attacks occurred, prompting gradual intensification of the regimen: duloxetine (a serotonin–norepinephrine reuptake inhibitor), buspirone (a non-benzodiazepine anxiolytic), and eszopiclone (a sedative-hypnotic) were added and titrated to target doses.

Acute cessation of panic attacks by hospital day 3 was primarily attributed to the discontinuation of albendazole combined with the fast-acting anxiolytic effect of alprazolam. Duloxetine and buspirone were initiated not for acute symptom relief — their therapeutic effects in panic disorder typically emerge after 2–4 weeks — but to address the persistent anticipatory anxiety, agoraphobic symptoms, and sleep disturbance that had developed during the 7-day exposure period. According to clinical practice guidelines for panic disorder, maintenance antidepressant and anxiolytic therapy is indicated to prevent symptom chronification and reduce recurrence risk in patients with established functional impairment.

The patient was discharged on November 19 on a stable oral medication schedule, with scheduled outpatient psychiatric follow-up for consolidation therapy and gradual tapering. No adverse effects of the psychotropic agents were observed during hospitalization.

## 3 Discussion

This case documents a probable albendazole-induced panic attack in a patient with recent PCI, underpinned by a Naranjo score of 8 (8). To our knowledge, this is the first report linking albendazole to a DSM-5-defined panic attack, thereby expanding the drug’s recognized neuropsychiatric adverse effect spectrum beyond the classic manifestations of encephalitis and demyelination (2–4).

### 3.1 Causality assessment

The temporal relationship was compelling: symptom onset within hours of the first dose, recurrence throughout the treatment course, and resolution after drug discontinuation combined with anxiolytic therapy. Although resolution was not immediate—consistent with the 8–12 hour elimination half-life of albendazole sulfoxide and potential tissue accumulation—the overall time course satisfies standard dechallenge criteria. Rechallenge was not performed, which is ethically appropriate given the severity of the reaction. Nevertheless, the 48-hour albendazole-free window after PCI discharge, during which the patient remained entirely asymptomatic on cardiovascular medications alone, serves as an informative negative-control period that strengthens the case for albendazole as the trigger. Alternative causes were systematically excluded: acute coronary syndrome, stent thrombosis, structural heart disease, pulmonary embolism, primary psychiatric disorder, and concomitant medications were all ruled out by appropriate investigations.

### 3.2 Biological plausibility

Albendazole sulfoxide, the active metabolite, is moderately lipophilic and can cross the blood–brain barrier, especially when the barrier is compromised (9). Benzimidazole derivatives have been shown to interfere with GABA-A receptor function, reducing inhibitory neurotransmission and increasing neuronal excitability—a mechanism implicated in both benzodiazepine withdrawal and experimental panic induction (9, 10). Disruption of serotonergic transmission may further dysregulate amygdala and hippocampal circuits involved in fear processing (10, 11).

Role of post-PCI state as a predisposing factor

In this patient, multiple concomitant factors may have acted synergistically to heighten central nervous system vulnerability, with recent PCI being the most prominent. Acute coronary syndrome and percutaneous stent implantation represent a major physiological and psychological stressor, inducing sustained sympathetic activation, increased interoceptive awareness, and heightened sensitivity to bodily sensations such as palpitations and chest discomfort. These adaptations lower the threshold for panic-like responses and create a permissive background for drug-induced anxiety symptoms. Additional contributing factors include untreated hyperglycemia and low-grade immune activation associated with chronic Clonorchis sinensis infection. Critically, however, the strict temporal relationship — symptom onset within hours of the first albendazole dose, and complete absence of anxiety symptoms during the 48-hour post-discharge period before albendazole initiation — strongly indicates that albendazole was the necessary and direct trigger, while these factors only increased background susceptibility.

Analysis of concomitant medications

All concurrent cardiovascular medications were assessed for their potential to induce panic attacks or interact with albendazole. Aspirin, clopidogrel, atorvastatin, sacubitril/valsartan, metoprolol succinate, and amlodipine have no established association with *de novo* panic attacks in the published literature. Notably, metoprolol succinate, a beta-adrenergic blocker, is commonly used to attenuate the somatic symptoms of anxiety and would be expected to reduce, rather than provoke, panic-like autonomic arousal. No clinically relevant pharmacokinetic interactions between these agents and albendazole have been reported that would increase central nervous system exposure to albendazole sulfoxide. Taken together, concomitant medications are unlikely to have contributed to the observed panic attacks.

### 3.3 Comparison with existing literature

Drug-induced panic attacks are uncommon and easily overlooked. The French pharmacovigilance analysis listed antidepressants, mefloquine, isotretinoin, and corticosteroids as main offenders; antiparasitic drugs were absent (5). Albendazole’s neurotoxicity typically manifests as encephalopathy (2, 3). Emerging evidence, however, points to a broader spectrum of neuropsychiatric effects: a large prospective cohort study found a 1.87-fold increased risk of chronic anxiety and depression in patients with neurocysticercosis treated with albendazole (12), and case reports have documented albendazole-induced acute psychosis (13, 14). All of these previous reports described chronic mood disturbances or psychotic features (hallucinations, delusions); none described isolated acute panic attacks.

### 3.4 Clinical implications

We propose two practical recommendations. First, clinicians should extend their vigilance for albendazole-related neuropsychiatric ADRs to include acute anxiety and panic-like presentations, particularly in patients with heightened central nervous system vulnerability (e.g., those recovering from major cardiovascular procedures, the elderly, or those with hepatic/renal impairment). In such populations, if the parasitic infection is not acutely life-threatening, deferring anthelmintic therapy until cardiovascular stability is achieved may be prudent. Second, patients prescribed albendazole should be informed of the potential for emergent anxiety or panic-like symptoms and advised to report them promptly. Early recognition, immediate drug discontinuation, and timely psychiatric referral can prevent progression from isolated panic attacks to panic disorder with agoraphobic avoidance, as observed in this case.

In conclusion, this case suggests that albendazole can induce acute panic attacks, likely via GABAergic and serotonergic dysregulation, especially in patients with heightened CNS vulnerability following major cardiovascular procedures. Three practical lessons emerge: first, clinicians should consider albendazole as a potential trigger for acute anxiety syndromes; second, a meticulous drug history and timeline are essential for differentiation; and third, early psychiatric referral can prevent progression to agoraphobia. Limitations of this single case include the absence of rechallenge, albendazole sulfoxide plasma levels, and CYP450 genotyping. Future pharmacovigilance studies are warranted to confirm this association.

### 3.5 Study strengths and limitations

#### 3.5.1 Strengths

This case report has several notable strengths. First, it documents, to our knowledge, the first DSM-5-defined acute panic attack attributed to albendazole, expanding the recognized neuropsychiatric adverse effect profile of this widely used anthelmintic. Second, causality was systematically assessed using the validated Naranjo scale, yielding a score of 8 that supports a probable causal relationship. Third, comprehensive diagnostic workup thoroughly excluded alternative organic and psychiatric causes. Fourth, the 48-hour drug-free observation period after PCI discharge provides an informative internal negative control, strengthening the temporal evidence for albendazole as the triggering agent. Finally, the report includes a detailed clinical timeline, patient perspective, and practical clinical recommendations, aligning with high-quality case reporting standards.

#### 3.5.2 Limitations

Several limitations should be acknowledged. First, as a single case report, the generalizability of the findings is limited, and the true incidence of albendazole-induced panic attacks cannot be estimated. Second, drug rechallenge was not performed, which is ethically justified given the severity of the reaction but prevents definitive confirmation of causality. Third, plasma concentrations of albendazole sulfoxide were not measured, so we cannot exclude individual pharmacokinetic variability as a contributing factor. Fourth, CYP450 genotyping was not available, which could have provided further insight into metabolic susceptibility. Fifth, the patient received combination psychotropic therapy, making it impossible to isolate the individual contribution of each agent to symptom resolution. Future large-scale pharmacovigilance studies are warranted to confirm this association and characterize risk factors.

## 4 Patient perspective

The patient described the episodes as “a sudden, overwhelming feeling that I was about to die or lose control of my mind.” After learning that his symptoms were likely a drug reaction rather than a new cardiac event, he expressed relief and hoped that sharing his experience might help other patients avoid similar distress. He also noted that the unpredictable nature of the attacks had made him afraid to be alone or to go out in public, which severely affected his daily life during that period.

## Data Availability

Restrictions apply to the availability of these clinical data to protect patient privacy. Therefore, the raw clinical data cannot be publicly shared. All relevant information supporting the findings is included within the article. Requests to access the datasets should be directed to the corresponding author.
