# Supplementary material for: Case Report: Panic attack induced by albendazole in a post-PCI patient
Source: Front Psychiatry. 2026 Aug 28;17:1915974. doi: 10.3389/fpsyt.2026.1915974 (PMC13562284; doi:10.3389/fpsyt.2026.1915974)
Supplement: Supplementary file 1 [file DataSheet1.pdf]

Topic	Item	Checklist item description	Reported on Line
Title	1	The diagnosis or intervention of primary focus followed by the words “case report”	Title, Line 1
Key Words	2	2 to 5 key words that identify diagnoses or interventions in this case report, including "case report"	Keywords section, between Author and Abstract
Abstract	3a	Introduction: What is unique about this case and what does it add to the scientific literature?	Abstract, Lines 10–13
Abstract	3b	Main symptoms and/or important clinical findings	Abstract, Lines 14–23
Abstract	3c	The main diagnoses, therapeutic interventions, and outcomes	Abstract, Lines 14–23
Abstract	3d	Conclusion—What is the main “take-away” lesson(s) from this case?	Abstract, Lines 24–28
Introduction	4	One or two paragraphs summarizing why this case is unique (may include references)	Introduction, Paragraph 2
Patient Information	5a	De-identified patient specific information	Case Presentation, Paragraph 1
Patient Information	5b	Primary concerns and symptoms of the patient	Case Presentation, Paragraphs 1–2
Patient Information	5c	Medical, family, and psycho-social history including relevant genetic information	Case Presentation, Paragraph 1 and Diagnostic Assessment
Patient Information	5d	Relevant past interventions with outcomes	Case Presentation, Paragraph 1
Clinical Findings	6	Describe significant physical examination (PE) and important clinical findings	Case Presentation, end of "Patient History and Initial Presentation"
Timeline	7	Historical and current information from this episode of care organized as a timeline	Table 1

Topic	Item	Checklist item description	Reported on Line
Diagnostic Assessment	8a	Diagnostic testing (such as PE, laboratory testing, imaging, surveys)	Diagnostic Assessment, Paragraph 1
Diagnostic Assessment	8b	Diagnostic challenges (such as access to testing, financial, or cultural)	Not applicable / Discussion, Limitation
Diagnostic Assessment	8c	Diagnosis (including other diagnoses considered)	Diagnostic Assessment, Paragraph 2
Diagnostic Assessment	8d	Prognosis (such as staging in oncology) where applicable	Not applicable
Therapeutic Intervention	9a	Types of therapeutic intervention (such as pharmacologic, surgical, preventive, self-care)	Therapeutic Intervention and Outcomes
Therapeutic Intervention	9b	Administration of therapeutic intervention (such as dosage, strength, duration)	Therapeutic Intervention and Outcomes
Therapeutic Intervention	9c	Changes in therapeutic intervention (with rationale)	Therapeutic Intervention and Outcomes
Follow-up and Outcomes	10a	Clinician and patient-assessed outcomes (if available)	Therapeutic Intervention and Outcomes
Follow-up and Outcomes	10b	Important follow-up diagnostic and other test results	Not explicitly mentioned, but follow-up is scheduled. Could also note "Not applicable"
Follow-up and Outcomes	10c	Intervention adherence and tolerability (How was this assessed?)	Therapeutic Intervention and Outcomes, last sentence
Follow-up and Outcomes	10d	Adverse and unanticipated events	Discussion, Causality assessment & Biological plausibility
Discussion	11a	A scientific discussion of the strengths AND limitations associated with this case report	Discussion, In conclusion paragraph
Discussion	11b	Discussion of the relevant medical literature with references	Discussion, Comparison with existing literature

Topic	Item	Checklist item description	Reported on Line
Discussion	11c	The scientific rationale for any conclusions (including assessment of possible causes)	Discussion, Causality assessment & Biological plausibility
Discussion	11d	The primary "take-away" lessons of this case report (without references) in a one paragraph conclusion	Discussion, In conclusion paragraph
Patient Perspective	12	The patient should share their perspective in one to two paragraphs on the treatment(s) they received	Patient Perspective section
Informed Consent	13	Did the patient give informed consent? Please provide if requested	Yes, Ethics Statement
